# The influence of math anxiety on symbolic and non-symbolic magnitude processing

**DOI:** 10.3389/fpsyg.2015.01621

**Published:** 2015-10-27

**Authors:** Julia F. Dietrich, Stefan Huber, Korbinian Moeller, Elise Klein

**Affiliations:** ^1^Leibniz-Institut für WissensmedienTuebingen, Germany; ^2^Department of Psychology, Eberhard Karls Universität TübingenTuebingen, Germany; ^3^Learning, Educational Achievement, and Life Course Development Graduate School, Eberhard Karls Universität TübingenTuebingen, Germany; ^4^Department of Neurology, Section Neuropsychology, University Hospital RWTH AachenAachen, Germany

**Keywords:** math anxiety, approximate number system, magnitude representation, symbolic, non-symbolic

## Abstract

Deficits in basic numerical abilities have been investigated repeatedly as potential risk factors of math anxiety. Previous research suggested that also a deficient approximate number system (ANS), which is discussed as being the foundation for later math abilities, underlies math anxiety. However, these studies examined this hypothesis by investigating ANS acuity using a symbolic number comparison task. Recent evidence questions the view that ANS acuity can be assessed using a symbolic number comparison task. To investigate whether there is an association between math anxiety and ANS acuity, we employed both a symbolic number comparison task and a non-symbolic dot comparison task, which is currently the standard task to assess ANS acuity. We replicated previous findings regarding the association between math anxiety and the symbolic distance effect for response times. High math anxious individuals showed a larger distance effect than less math anxious individuals. However, our results revealed no association between math anxiety and ANS acuity assessed using a non-symbolic dot comparison task. Thus, our results did not provide evidence for the hypothesis that a deficient ANS underlies math anxiety. Therefore, we propose that a deficient ANS does not constitute a risk factor for the development of math anxiety. Moreover, our results suggest that previous interpretations regarding the interaction of math anxiety and the symbolic distance effect have to be updated. We suggest that impaired number comparison processes in high math anxious individuals might account for the results rather than deficient ANS representations. Finally, impaired number comparison processes might constitute a risk factor for the development of math anxiety. Implications for current models regarding the origins of math anxiety are discussed.

## Introduction

Mathematics anxiety has been defined as feelings of tension, apprehension, or fear which interfere with math performance in various contexts such as school but also everyday and professional life (e.g., Richardson and Suinn, 1972; Ashcraft, 2002). Thus, negative consequences of math anxiety are serious and far-reaching. On the psychological level, math anxiety was found to be associated with reduced motivation for math and lower self-concept as regards math (Hembree, 1990). On the behavioral level, math anxiety was related to a tendency to avoid mathematics (Hembree, 1990). Additionally, math anxiety was also found to be associated negatively with math performance (see Hembree, 1990; Ma, 1999, for meta-analyses). This latter point is particularly worrying, because numerical abilities are key competences in our society today, which predict individual scholastic and professional prospects (Bynner and Parsons, 2006; Hudson et al., 2009). Due to this wide range of negative effects associated with math anxiety, it is important to understand the factors leading to the development of math anxiety.

Currently, there are several approaches accounting for the development of mathematics anxiety (e.g., see Maloney and Beilock, 2012, for a review). The most comprehensive model by Ashcraft et al. (2007) postulates three risk factors for the development of math anxiety: (1) inadequate math skills, (2) insufficient motivation, or (3) poor working memory. All these three factors can lead to deficits in math performance which increase the probability of developing math anxiety (see also Ashcraft and Moore, 2009 for a detailed description of the model). In combination with negative learning experiences (e.g., negative feedback of teachers and parents) these risk factors may also lead to negative attitudes toward math and increase self-focused attention and rumination, which in turn may contribute to the development of math anxiety.

Ashcraft and Moore (2009) proposed that the risk factor ‘inadequate math skills’ may also include deficits in basic numerical competencies such as counting or number knowledge. In line with this, Maloney et al. (2010) found that high math anxious individuals indeed presented with deficient counting abilities. Moreover, Maloney et al. (2011) proposed that a deficient representation of numerical magnitude (i.e., a deficient approximate number system; ANS) might contribute to the development of math anxiety (see also Núñez-Peña and Suárez-Pellicioni, 2014). The ANS is assumed to represent numerical magnitude information (i.e., numerosity) in an approximate manner (e.g., Cantlon et al., 2009). In particular, it was suggested that numerosities are represented in the ANS by overlapping Gaussian tuning curves. These tuning curves reflect the activity of neurons showing a maximum neural activation for a specific magnitude and an attenuated activation for adjacent magnitudes (e.g., see Feigenson et al., 2004, for a review). Importantly, the ANS was proposed to constitute a building block for later more complex numerical/mathematical abilities (Dehaene, 2001). In line with this, ANS acuity was found to predict later math performance (e.g., Mazzocco et al., 2011; Libertus et al., 2013).

Supporting the idea of math anxiety being caused by a deficient ANS, Maloney et al. (2011) found a larger distance effect for high math anxious individuals than for low math anxious individuals (see Núñez-Peña and Suárez-Pellicioni, 2014, for similar results). The distance effect describes the finding that response time (RT) and error rates (ERs) increase as the numerical distance between two to-be-compared numbers decreases (Moyer and Landauer, 1967). For instance, participants’ RTs and ERs are larger when comparing 3 vs. 4 than when comparing 2 vs. 8. This effect can be explained by a larger overlap of the ANS representations for less distant magnitudes according to ANS theory (e.g., Dehaene et al., 1998). Additionally, Núñez-Peña and Suárez-Pellicioni (2014) observed a marginal significant interaction between the size effect and math anxiety. In their study, high math anxious participants showed a larger size effect than low math anxious participants. The size effect refers to the observation that the processing of numbers becomes more difficult as the size of the numbers to be processed increases (see Zbrodoff and Logan, 2005, for a review). Also the size effect can be explained by ANS theory (e.g., Dehaene et al., 1998). It is assumed that the overlap between the ANS representations increases with numerosity (Feigenson et al., 2004). Thus, it should be more difficult to discriminate between larger magnitudes (e.g., 8 vs. 9) than between smaller magnitudes (e.g., 1 vs. 2; i.e., the size effect, e.g., Parkman, 1971).

Both studies assessed ANS acuity using a symbolic Arabic number comparison task (Maloney et al., 2011; Núñez-Peña and Suárez-Pellicioni, 2014). However, this procedure was based on the common assumption that magnitude representations in the ANS are abstract, this means modality independent (e.g., Libertus et al., 2007; Piazza et al., 2007). Hence, the ANS may be assessed using either Arabic number symbols or non-symbolic stimuli such as dot patterns. However, there is accumulating evidence questioning the notion of an abstract, modality-independent magnitude representation (see Cohen Kadosh and Walsh, 2009 for a review). Only recently, for example, Bulthé et al. (2014) found no representational overlap for symbolic and non-symbolic magnitudes. Moreover, Lyons et al. (2015) showed that symbolic and non-symbolic magnitudes are coded fundamentally differently. These recent results question the assumption that ANS acuity may be measured validly using a symbolic number comparison task. This in turn challenges the conclusion of Maloney et al. (2011; see also Núñez-Peña and Suárez-Pellicioni, 2014) that the association between math anxiety and the symbolic distance effect found in previous studies should be driven by a deficient ANS.

Moreover, it was also questioned recently whether the distance and/or the size effect – derived from a symbolic number magnitude comparison task – are valid indices of ANS acuity (e.g., Verguts et al., 2005; Van Opstal et al., 2008). For instance, Van Opstal et al. (2008) observed that the distance effect measured in a symbolic number magnitude comparison task does not necessarily imply an overlap of the magnitude representations of individual numbers as suggested by the ANS theory. Instead, the distance effect might be driven by response-related processes. Furthermore, Verguts et al. (2005) provided an alternative explanation for the size effect. In a computational modeling study, they showed that the size effect depended on the differential frequency of the individual numbers during learning (i.e., the lower frequency of larger numbers). In line with this result, Dehaene and Mehler (1992) found that the frequency of numbers in daily life decreased with increasing numerical magnitude, which might cause the size effect. These alternative explanations for the distance and size effect further question the conclusions of previous studies that modulations of the distance effect and the size effect by math anxiety are associated with ANS acuity, because both effects may not necessarily reflect ANS acuity.

Hence, in the present study, we examined whether ANS acuity is indeed related to math anxiety and, consequently, may represent a risk factor for the development of math anxiety. To do so, we employed the current standard task to assess ANS acuity: the non-symbolic dot comparison task (see De Smedt et al., 2013; Dietrich et al., 2015, for reviews). Recently, there has been an increasing amount of research concentrating on cognitive processes involved in the solution process of a dot comparison task (e.g., inhibitory control, Gilmore et al., 2013) and methodological factors influencing task performance. For instance, task performance was found to be influenced by visual stimulus parameters (e.g., Szűcs et al., 2013), presentation duration (Inglis and Gilmore, 2013) and set size (Clayton and Gilmore, 2015; see Dietrich et al., 2015, for a detailed discussion about the reliability/validity of ANS tasks as well as methodological aspects relevant for the design of an ANS task). Therefore, we designed the non-symbolic dot comparison task considering these methodological aspects in order to assess ANS acuity as reliably and as validly as possible. Moreover, several measures were used to index ANS acuity. However, recent studies question the assumption that all indices can be used interchangeably, although this issue is not fully resolved yet (Lindskog et al., 2013; Inglis and Gilmore, 2014). To account for this methodological issue we considered several indices to reflect ANS acuity: ER, mean RT, the distance, the size effect as well as the Weber fraction. The latter is assumed to be the most direct index of ANS acuity (reflecting the width of the Gaussian tuning curves, i.e., the precision of the ANS representations; Pica et al., 2004). If a deficient ANS indeed underlies math anxiety, this should be reflected by a reliable association of math anxiety and these measures. However, a significant correlation might be present not for all measures, because previous research found considerable differences regarding the reliability of these measures (Lindskog et al., 2013; Inglis and Gilmore, 2014). To allow for a direct comparison of our results with previous findings, we also administered a symbolic number comparison task. We expected to replicate the findings of Maloney et al. (2011) and Núñez-Peña and Suárez-Pellicioni (2014) who found a (marginally) significant positive association between math anxiety and the distance and the size effect for RTs in the symbolic number magnitude comparison task.

## Materials and Methods

### Participants

Sixty-one undergraduates (37 female, 3 left-handed, mean age = 24.7 years, *SD* = 3.3 years) participated in the experiment. All participants were informed about the experimental procedure before they provided written consent to participate. Participation was compensated with 8AAA per hour. The study was approved by the local ethics committee of the Leibniz-Institut für Wissensmedien.

### Materials

#### The Abbreviated Math Anxiety Scale (AMAS)

The AMAS is a nine-item scale to assess math anxiety. Participants are asked to indicate on a 5-point Likert scale from 1 (low anxiety) to 5 (high anxiety) how anxious they feel in various math-related situations. Adequate internal consistency (Cronbach’s α = 0.90), test-retest reliability (*r* = 0.85) and construct validity have been reported for this instrument (Hopko et al., 2003). In the current study the internal consistency of the AMAS was similar to the results of Hopko et al. (2003; Cronbach’s α = 0.92). The AMAS score was calculated by adding up participants responses on the Likert scales.

#### Symbolic Number Comparison

In the symbolic number comparison task, two single digits were presented simultaneously, one above the other on a 19 inch monitor with a resolution of 1024 pixel × 768 pixel and 75 Hz. Participants had to single out the larger of the two digits. When the upper digit was the larger one, they should press the “Z” key on a standard QWERTZ keyboard with their right index finger. When the lower digit was the larger one, they should press the “B” key with their left index finger. The digits remained visible on the screen until the participants pressed one of the response buttons. Each number pair was preceded by a fixation point, which was presented for 500 ms. All possible combinations of the single digits 1 to 9 (i.e., 72 different digits pairs) were presented five times resulting in a total of 360 experimental trials. Participants completed five practice trials before the experimental trials started. Digits were presented using font “Courier New” with font size set to 60 at the x/y coordinates 512/484 and 512/284. The internal consistency of the symbolic number comparison task was Cronbach’s α = 0.92 and Spearman-Brown corrected split-half reliability was *r* = 0.87.

#### Non-symbolic Dot Comparison

In the non-symbolic dot comparison task, two dot sets were presented simultaneously – one set on the left and one set on the right side of the screen. Both sets were separated by a black vertical line. Participants were instructed to indicate, which of the two sets contained more dots, by pressing the corresponding key (i.e., press the left Ctrl key when the left set is larger or the right Ctrl key when the right set is larger). Position of the larger dot set was counterbalanced across screen sides. Dot sets included black dots against a white background and were presented on the screen for 200 ms. Afterward a white screen was presented, which remained visible until participants pressed one of the response keys. Each trial started with a fixation sign (i.e., a black square) displayed for 500 ms. Dot sets contained between 10 and 40 dots. The ratios between the two to-be compared dot sets were 0.5, 0.6, 0.7, 0.8, and 0.9. There were 80 trials per ratio resulting in a total of 400 experimental trials. Before the experimental trials started, five practice trials were presented. To control for visual properties, dot sets were created with the MATLAB script of Gebuis and Reynvoet (2011). In half of the trials convex hull (i.e., area in which the dots can appear) and item size (i.e., average diameter of the dots in one set) were larger for the more numerous set, whereas in the other half of the trials, convex hull and item size were smaller for the more numerous set. The internal consistency of the non-symbolic dot comparison task was Cronbach’s α = 0.96 and Spearman-Brown corrected split-half reliability was *r* = 0.91.

### Procedures

All participants were tested individually. First, they had to complete the AMAS followed by the magnitude comparison tasks. The order of the symbolic number comparison task and the non-symbolic dot comparison task was counterbalanced across participants.

### Analysis

We analyzed RTs as well as ERs. We included all responses in the analysis of RTs (correct and incorrect responses). This procedure was chosen, because in the non-symbolic comparison task the percentage of errors was quite high, which would have reduced the number of observations considerably. However, the same analyses including only RTs of correct responses did yield the same pattern of results. A trimming procedure excluded RTs deviating more than 3 *SD* from the individual mean. This outlier analysis reduced the data set of the symbolic number comparison task by 1.63% and the data set of the non-symbolic dot comparison task by 1.77%.

Response times were analyzed using linear mixed effects models (LME). For the analysis of ER, generalized linear mixed effects models (GLME) with a binomial error distribution and the logit as link function were employed. All statistical analysis were run using R (R Core Team, 2015) and the R package lme4 for the (G)LME (Bates et al., 2014). The *p*-values for LME were calculated using the Satterthwaite approximation for degrees of freedom available via the R package lmerTest (Kuznetsova et al., 2015). The *p*-values for GLME were derived via likelihood ratio tests using the R package afex (Singmann et al., 2015).

Fixed effects in our analyses (LME and GLME) were distance (i.e., the distance between the to-be-compared numbers/numerosities), size (i.e., the sum of the two numbers/dots in both sets), the AMAS score and the interaction between distance and AMAS score as well as between size and AMAS score. The predictors distance and size were z-transformed prior to data analysis and the AMAS score was centered.

In line with the suggestion of Barr et al. (2013), we first attempted to fit the LME for RT data using the maximum random effects structure. Thus, we included the fixed effects distance and size also as random effects as well as a random intercept for participants in the analysis of the symbolic and the non-symbolic comparison task and a random intercept for items in the analysis of the non-symbolic comparison task. In the GLME (ER data) for the symbolic comparison task, we included a random intercept for participants. In the GLME (ER data) for the non-symbolic comparison task, we also included a random intercept for items to account for the fact that we included only a sample of all possible items.

Additionally, we estimated the Weber fraction indicating the acuity of the ANS representation using the following formula (Pica et al., 2004):

$$f_{acc}\left(r,\;w\right)\,\;=\;1\,\;-\;\frac{1}{2}erfc\left(\frac{\left|r-1\right|}{\sqrt{2w}\sqrt{r^{2}+1}}\right)$$

The formula describes the probability *f_acc_* of correctly comparing two numerosities with a given ratio *r* (i.e., the ratio between the larger and the smaller numerosity) for a participant with an internal Weber fraction *w* using the complementary Gauss error function erfc. Individual Weber fractions were fitted using the Gauss–Newton algorithm for non-linear least squares fit on the mean accuracy for each ratio and the R package pracma for the erfc function (Borchers, 2015). For eight participants, the fitting function did not converge or the Weber fraction was not a reliable predictor of mean accuracy of participants. Thus, we included 53 participants in the analysis containing the Weber fraction. To investigate, whether math anxiety is related to ANS acuity indexed by the Weber fraction we conducted a linear regression analysis with AMAS score as dependent variable and the individual Weber fraction as independent variable. Null effects were validated using a Bayesian model selection approach, which investigates whether the null hypothesis or the alternative hypothesis is more supported by the data (Masson, 2011). We calculated the posterior probability that the data favor the null hypothesis and the complement that the data favor the alternative hypothesis. According to the classification of Raftery (1995) a posterior probability of >0.75 provides positive evidence in favor of the investigated hypothesis.

## Results

An overview of the descriptive statistics of the variables is given in **Table 1.** Additionally, **Table 2** shows the relationships between all these variables.

**Table 1:** Mean, standard deviation (*SD*) and range of all variables.

Variable	Mean	*SD*	Minimum	Maximum
Abbreviated Math Anxiety Scale (AMAS) score	22.03	8.11	10	39
ER symbolic comparison task	3.82%	2.95%	0.28%	16.94%
ER non-symbolic comparison task	32.92%	9.02%	17.25%	50.75%
RT symbolic comparison task	664.75 ms	80.64 ms	514.45 ms	899.44 ms
RT non-symbolic comparison task	681.87 ms	199.22 ms	304.52 ms	1278.52 ms
Weber fraction	0.60	0.40	0.24	2.74


*Higher AMAS scores reflect higher math anxiety and vice versa. Smaller Weber fractions reflect a more precise ANS. ER, error rate; RT, response time.*

**Table 2:** Spearman correlation coefficients between all variables.

	(1)	(2)	(3)	(4)	(5)	(6)
(1) AMAS score	1					
(2) ER symbolic comparison task	-0.10	1				
(3) RT symbolic comparison task	0.16	-0.53^∗∗∗^	1			
(4) ER non-symbolic comparison task	0.11	0.13	0.16	1		
(5) RT non-symbolic comparison task	-0.10	-0.37^∗^	0.39^∗^	-0.38^∗^	1	
(6) Weber fraction	0.09	0.13	0.18	0.99^∗∗∗^	-0.36^∗^	1


*^∗^p < 0.05, ^∗∗∗^p < 0.001; p-values were adjusted for multiple testing using the Benjamini-Hochberg procedure (Benjamini and Hochberg, 1995). ER, error rate; RT, response time; AMAS, Abbreviated Math Anxiety Scale.*

### Response Times

**FIGURE 1:**
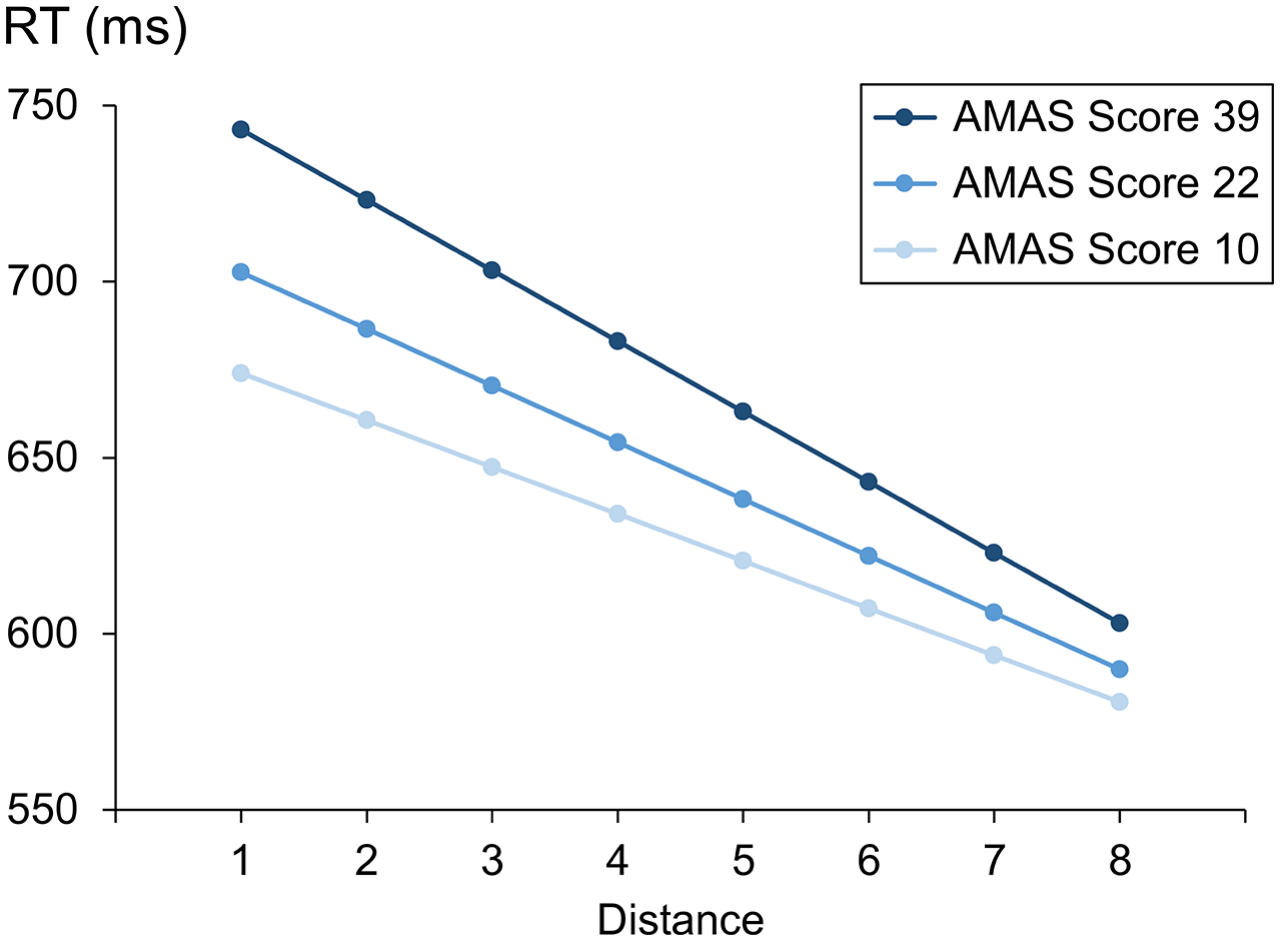
**Illustration of the estimated distance effects for participants with low math anxiety [Abbreviated Math Anxiety Scale (AMAS) score = 10, the smallest AMAS score in our sample], middle math anxiety (AMAS score = 22, mean of AMAS scores in our sample) and high math anxiety (AMAS score = 39, the largest AMAS score in our sample)**.

**FIGURE 2:**
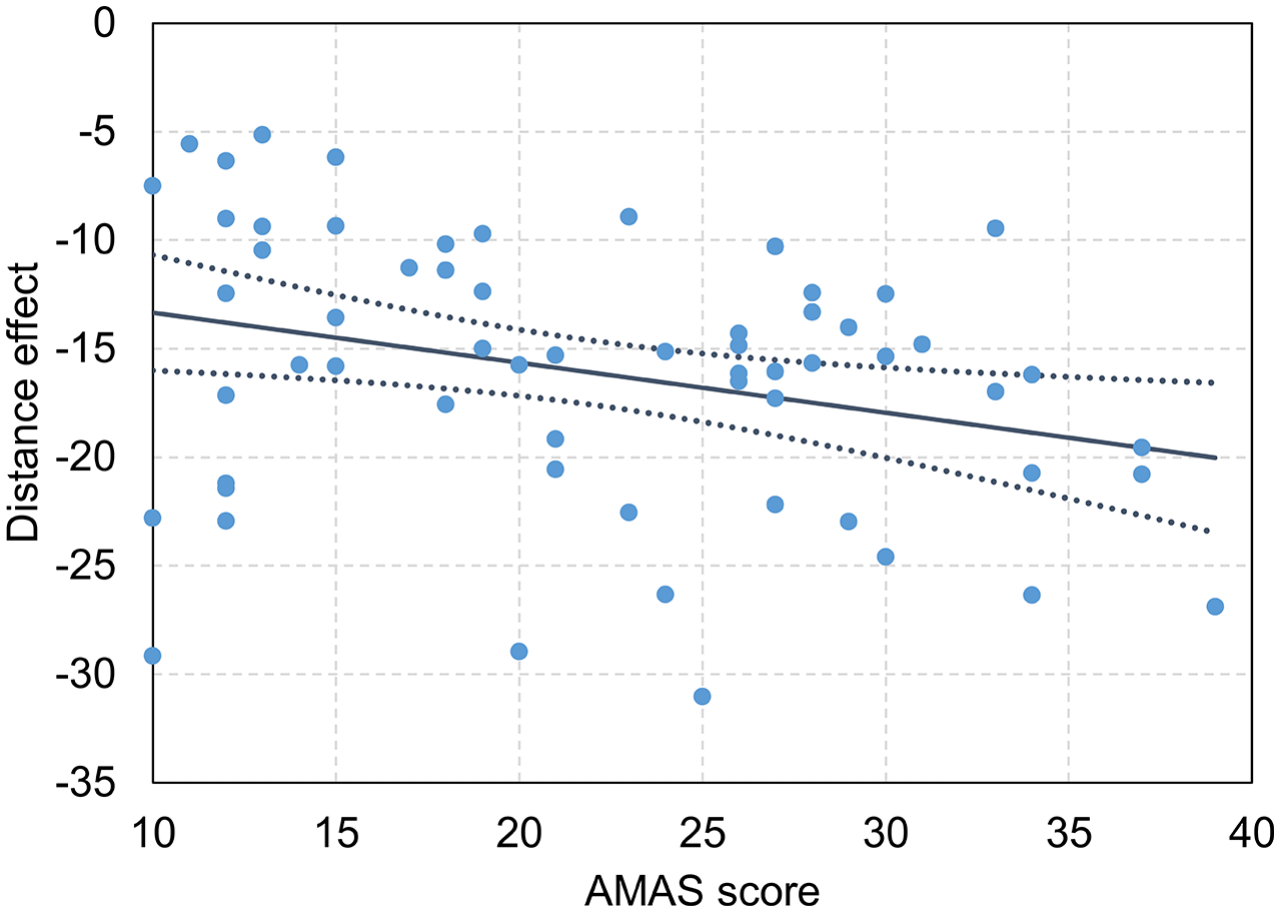
**Relationship between symbolic distance effect for response times (RTs) and Abbreviated Math Anxiety Scale (AMAS) score.** Dark blue line reflects slope of the interaction between distance effect and AMAS score, dotted dark blue lines indicates 95% CI, dots reflect individual distance effects and AMAS scores. Higher AMAS scores reflect higher math anxiety and vice versa.

First, we investigated separately for the symbolic and the non-symbolic comparison task whether math anxiety (reflected by the AMAS score) influences overall RT as well as the distance and the size effect for RT. A possible influence of math anxiety on overall RT would be indicated by a significant main effect of the AMAS score. Furthermore, an influence of math anxiety on distance or size effects for RT would be reflected by a significant interaction between AMAS score and distance or size. The results of the LME for RT data are given in **Table 3**, both for the symbolic number comparison task and the non-symbolic dot comparison task. We observed reliable effects of numerical distance and size for both tasks. For the symbolic comparison task, RT decreased with numerical distance between the two digits and increased with their numerical size. Similarly, the significant distance effect in the non-symbolic dot comparison task indicated that participants’ RT decreased with numerical distance between the two dot sets. However, in contrast to the symbolic comparison task, we found that RT decreased with numerical size for the non-symbolic dot comparison task. Importantly, we observed a reliable interaction between AMAS score and distance in the symbolic comparison task. As shown in **Figures 1** and **2**, the interaction indicated that participants with a higher AMAS score showed a larger distance effect than participants with a lower AMAS score. However, we did not find a significant interaction between AMAS score and distance in the non-symbolic dot comparison task. For both, the symbolic and the non-symbolic task there was no significant interaction between size and AMAS score. Moreover, there was also no reliable effect of the AMAS score on RT. An analog analysis with a categorical predictor (i.e., low vs. high math anxious group) instead of a continuous predictor for the AMAS score revealed an identical pattern of results (see Table A1 in the Supplemental Material).

**Table 3:** Estimates of fixed effects (ms) for response times.

Task	Effect	Estimate (*SE*)	df	*t*	*p*	95% CI
Symbolic comparison	Intercept	665.063 (10.07)	61.00	66.03	<0.001	[645.32, 684.80]
	Distance	-16.111 (0.75)	60.99	-21.36	<0.001	[-17.59, -14.63]
	AMAS	1.848 (1.25)	61.00	1.48	0.145	[-0.61, 4.30]
	Size	4.157 (0.44)	60.90	9.52	<0.001	[3.30, 5.01]
	Distance × AMAS	-0.230 (0.09)	60.99	-2.46	0.017	[-0.41, -0.05]
	Size × AMAS	0.040 (0.05)	60.85	0.74	0.461	[-0.07, 0.15]
Non-symbolic comparison	Intercept	682.048 (25.28)	61.35	26.99	<0.001	[632.51, 731.59]
	Distance	-1.876 (0.57)	93.23	-3.28	0.001	[-3.00, -0.75]
	AMAS	-1.880 (3.14)	61.00	-0.60	0.551	[-8.03, 4.27]
	Size	-1.117 (0.22)	98.06	-5.13	<0.001	[-1.54, -0.69]
	Distance × AMAS	-0.056 (0.06)	60.79	-0.88	0.380	[-0.18, 0.07]
	Size × AMAS	-0.004 (0.02)	60.85	-0.18	0.859	[-0.05, 0.04]


*95% CI based on the estimated local curvature of the likelihood surface.*

### Error Rates

Second, similar to the analysis for RT, we investigated the influence of math anxiety (i.e., AMAS score) on overall performance as well as distance and size effects based on ERs. Again, an influence of math anxiety would be indicated by either a significant main effect of AMAS score or a reliable interaction between AMAS score and distance or size. The results for ER data are summarized in **Table 4.** In line with the results for RT, we found reliable distance and size effects for both tasks. For the symbolic task, we observed that ER decreased as the numerical distance between the numbers increased (log odds = -0.349; in %: -0.64%), whereas they increased with the size of the numbers (log odds = 0.148; in %: 0.34%). The same pattern was observed for the non-symbolic task. ER also decreased with the numerical distance between dot sets (log odds = -0.070; in %: -1.45%) and increased with their size (log odds = 0.009; in %: 0.18%). There were no significant interactions between the AMAS score and distance or size neither in the symbolic nor in the non-symbolic task. Hence, we could not find an analog pattern for ER as for RT, where we found a significant interaction between the AMAS score and the distance effect for the symbolic comparison task. The missing interaction for ER might be explained by a ceiling effect for the symbolic comparison task. The ERs were very low, which might have reduced the variance and, hence, the effect. Moreover, there was no significant effect of the AMAS score on ER. An analog analysis with a categorical predictor for the AMAS score revealed an identical pattern of results (see Table A2 in the Supplemental Material).

**Table 4:** Estimates of fixed effects (log odds) for error rates.

Task	Effect	Estimate (*SE*)	χ^2^	*p*	95% CI
Symbolic comparison	Intercept	-3.788 (0.107)	-	-	[-3.998, -3.579]
	Distance	-0.349 (0.244)	242.58	<0.001	[-0.397, -0.302]
	AMAS	-0.012 (0.013)	0.83	0.363	[-0.038, 0.014]
	Size	0.148 (0.010)	234.34	<0.001	[0.128, 0.168]
	Distance × AMAS	0.001 (0.003)	0.03	0.868	[-0.006, 0.007]
	Size × AMAS	<0.001 (0.001)	0.01	0.926	[-0.002, 0.003]
Non-symbolic comparison	Intercept	-0.840 (0.071)	-	-	[-0.980, -0.700]
	Distance	-0.070 (0.008)	70.18	<0.001	[-0.085, -0.054]
	AMAS	0.005 (0.008)	0.47	0.493	[-0.010, 0.020]
	Size	0.009 (0.003)	7.21	0.007	[0.002, 0.015]
	Distance × AMAS	<0.001 (<0.001)	0.05	0.818	[-0.001, 0.001]
	Size × AMAS	<0.001 (<0.001)	1.56	0.212	[>-0.001, <0.001]


*P-values were obtained via likelihood ratio tests (df = 1). 95% CI are based on the estimated local curvature of the likelihood surface.*

### Weber Fraction

Finally, we investigated whether the Weber fraction, which is assumed to be the most direct measure of ANS acuity, was related to the individual AMAS score. A linear regression analysis predicting AMAS score from individual Weber fraction revealed no significant effect [*B* = 3.119, β = 0.148, *t*(51) = 1.07, *p* = 0.292]. Moreover, the model accounted for only 2.18% of the variance in AMAS score [*F*(1,51) = 1.14, *p* = 0.292]. This null effect was further investigated using a Bayesian model selection approach. The posterior probability for the null hypothesis was 0.80 providing positive evidence for the null hypothesis (i.e., no relationship between AMAS score and Weber fraction) according to Raftery (1995).

## Discussion

In the present study, we investigated whether ANS acuity is related to math anxiety and may, therefore, constitute a risk factor for the development of math anxiety. Complementing previous studies (Maloney et al., 2011; Núñez-Peña and Suárez-Pellicioni, 2014) we used not only a symbolic number comparison task but also a non-symbolic dot comparison task to assess ANS acuity. Additionally, we employed not only the distance and the size effect as indices of ANS acuity, but also evaluated ER, mean RT, and the Weber fraction. The latter is assumed to reflect the precision of the ANS representations directly (e.g., Pica et al., 2004). We replicated the significant association between math anxiety and the distance effect based on RT for the symbolic number comparison task. However, we did not observe an association of size effect and math anxiety. Furthermore, we did not observe a relationship between math anxiety and any of the ANS measures based on the non-symbolic dot comparison task. In the following, we will first discuss the implications of these results for the proposed association of ANS acuity and math anxiety before elaborating conclusions for symbolic number processing and math anxiety and theoretical implications for models on the origins of math anxiety.

### ANS Acuity and Math Anxiety

Recently, it was suggested that a less precise ANS might contribute to the development of math anxiety (Maloney et al., 2011; Núñez-Peña and Suárez-Pellicioni, 2014). These studies, however, did not measure ANS acuity using a non-symbolic dot comparison task, which represents the standard task to assess ANS acuity (e.g., Halberda et al., 2008; De Smedt et al., 2013; Gilmore et al., 2014; Inglis and Gilmore, 2014; Dietrich et al., 2015), but used a symbolic number comparison task instead. The use of the symbolic number comparison task to assess ANS acuity is valid when assuming that numerical magnitudes are represented in the ANS in an abstract, modality-independent manner. In this case only ANS acuity can be assessed using either symbolic or non-symbolic magnitude comparison tasks. However, recent studies challenged the assumption of such an abstract representation of numerical magnitude (Bulthé et al., 2014; Lyons et al., 2015), and therewith also question conclusions regarding the association of ANS acuity and math anxiety reported so far.

The ANS is assumed to support the comparison and estimation of numerosities (Dehaene, 2001, 2009) and should, therefore, be involved in the solution of a dot comparison task. Importantly, evidence for this assumption was provided by numerous studies with several methodological approaches. Single-cell recordings with monkeys revealed numerosity-selective neurons in the prefrontal and intraparietal cortex responding with a maximum activity to a specific numerosity (i.e., number of dots in a set; Nieder et al., 2002; Nieder, 2012; see also Ditz and Nieder, 2015, for a similar finding in songbirds). However, the neurons fired not exclusively for a specific numerosity, but they were also but less activated by adjacent numerosities. This pattern fitted well to the postulated overlapping Gaussian tuning curves of ANS representations, which increase in their width (i.e., imprecision) as the numerosities increase (Nieder, 2005). Further evidence comes from human brain-imaging studies (e.g., Piazza et al., 2004, 2007; Harvey et al., 2013). For instance, in line with ANS theory Lyons et al. (2015) showed that non-symbolic numerosities are represented by overlapping tuning curves, whereby the neuronal overlap increases with increasing numerosities. Moreover, the pattern of overlapping ANS representations was also reflected by behavioral performance in humans in a delayed match-to-sample task, as the percentage to judge a numerosity matching a sample was highest for the exact match and decreased as the distance between the numerosity of the stimulus and the sample increased (Merten and Nieder, 2009). Hence, several studies evaluating the validity of dot comparison tasks provided conclusive evidence that the dot comparison task assesses ANS acuity (both on a neuronal and a behavioral level). Nevertheless, there are also studies indicating that other cognitive processes are involved in the dot comparison task (e.g., inhibitory control, Fuhs and McNeil, 2013; Gilmore et al., 2013; Clayton and Gilmore, 2015). Moreover, the performance in the dot comparison task was found to be influenced by methodological aspects (e.g., task design, Price et al., 2012; presentation duration of the stimuli, Inglis and Gilmore, 2013; visual parameters, Szűcs et al., 2013). However, our results support the view that the non-symbolic dot comparison task used in our study (also) assessed ANS acuity, as we found both a significant distance and size effect. These effects are considered a result of the imprecise ANS representations and so far there are no alternative explanations for the occurrence of a distance or size effect in non-symbolic comparison tasks. Hence, the distance/ size effects indicate that the ANS was involved in the solution of the task (e.g., Dehaene et al., 1998). Additionally, we were able to fit the Weber fraction to the results of a vast majority of the participants. The Weber fraction is assumed to directly reflect the width of the ANS representations (Pica et al., 2004). Using a non-symbolic dot comparison task, we did not observe a significant association between several indices of ANS acuity and math anxiety. Thus, ANS acuity was not impaired in individuals being more math anxious. Importantly, we not only used the distance and size effect as measures of ANS acuity but also the Weber fraction, which is thought to be the most direct measure of the ANS acuity (Pica et al., 2004). However, comparable to the results for the distance and the size effect, which were already used as measures of ANS acuity in previous studies on the relationship between ANS acuity and math anxiety, we did not find an association between the Weber fraction and math anxiety as well. Moreover, also our Bayesian analysis revealed positive evidence for the null hypothesis.

Taken together, we did not find a reliable association between ANS acuity and math anxiety – independent of the measure used to assess ANS acuity. Therefore, our results are not in line with the conclusion of previous studies (Maloney et al., 2011; Núñez-Peña and Suárez-Pellicioni, 2014) that low ANS acuity is related to and may thus contribute to the development of math anxiety.

### Symbolic Number Comparison and Math Anxiety

Our results suggest that ANS acuity does not seem to be related to math anxiety. This raises the question of how to interpret previous and the present results revealing an association of the symbolic distance effect (or size effect) and math anxiety (Maloney et al., 2011; Núñez-Peña and Suárez-Pellicioni, 2014). So far, these results have been explained by less precise magnitude representations in the ANS. However, our results based on the non-symbolic dot comparison task revealed no association between the acuity of the ANS and math anxiety questioning this explanation.

We did not find an overall relationship between math anxiety and performance (i.e., RT and ER) in the symbolic number comparison task. Thus, high math anxious individuals did not *per se* perform worse and/or slower than less math anxious individuals. However, we found a significant association of the distance effect based on RT and math anxiety replicating the findings of previous studies (Maloney et al., 2011; Núñez-Peña and Suárez-Pellicioni, 2014). Higher math anxious individuals presented with a larger distance effect than those with lower math anxiety. As we did not find a relationship between ANS acuity and math anxiety, this effect cannot be interpreted as being due to less precise magnitude representations in the ANS. Thus, this finding has to be reinterpreted.

There is evidence that the distance effect for symbolic number comparison can be explained by comparison processes (i.e., the connection between the symbolic representation and the response, Van Opstal et al., 2008). In line with this explanation for the distance effect in symbolic number comparison, the association between the distance effect in symbolic number comparison and math anxiety might be due to impaired comparison processes rather than impaired magnitude representations in high math anxious individuals. The connection between the representation and the “which numeral is larger” response might weaker be due to less training of this connection, for example, when math anxious children are not motivated to operate with numbers or avoid working with numbers.

In the present study, we did not find a significant interaction between the size effect for RT and math anxiety. Núñez-Peña and Suárez-Pellicioni (2014) found a tendency for a larger size effect in high math anxious individuals compared to low math anxious individuals. Moreover, compared to our results, the size effect was generally larger in the study of Núñez-Peña and Suárez-Pellicioni (2014). These differences might be due to differences in the design. First, Núñez-Peña and Suárez-Pellicioni (2014) instructed the participants to respond as fast as possible, whereas in the present study the instruction stressed not only speed but also accuracy. Second, in the study by Núñez-Peña and Suárez-Pellicioni (2014) symbolic stimuli were presented for only 300 ms, whereas in the present study stimuli remained visible until a response was given. These two aspects might have induced larger variance in the responses observed by Núñez-Peña and Suárez-Pellicioni (2014), which in turn might have resulted in a larger size effect allowing for a better chance to find a (marginally) significant association of the size effect and math anxiety.

### Theoretical Implications

From a theoretical point of view, our results allow for a specification of the model by Ashcraft et al. (2007) who postulated that inadequate basic numerical competencies might constitute a risk factor for the development of math anxiety (Ashcraft and Moore, 2009). According to our findings this risk factor might include deficits in symbolic number comparison. More precisely, our results indicate that comparison processes seem to be impaired in high math anxious individuals, because math anxiety was associated with the symbolic distance effect (Van Opstal et al., 2008). Further evidence for our conclusion that deficits in symbolic number comparison might indeed constitute a risk factor for the development of math anxiety [as suggested by Ashcraft et al. (2007)] comes from studies indicating a general relationship between the distance effect in symbolic number comparison and math performance (e.g., Holloway and Ansari, 2009). One mechanism for the development of math anxiety according to the model of Ashcraft et al. (2007) is that inadequate math skills lead to math performance deficits, which in turn support the development of math anxiety. Thus, deficits in basic numerical abilities such as the comparison of symbolic numbers should be associated with lower math performance. In line with this suggestion De Smedt et al. (2009) found that the symbolic distance effect for RTs predicted later math performance, whereby a larger distance effect was associated with lower later math performance. In turn, according to the model of Ashcraft et al. (2007) lower math performance contributes to the development of math anxiety. And thus, a more pronounced distance effect should be associated with higher math anxiety, which is exactly what we found (see also Maloney et al., 2011; Núñez-Peña and Suárez-Pellicioni, 2014).

However, it remains an open question what causes the larger symbolic distance effect in more math anxious individuals. When interpreting this effect as impaired comparison processes this might be explained by less trained connections between the symbolic representation of the number and the response. This finding might be due to an insufficient motivation of the children to work with the numbers. Insufficient motivation is another risk factor according to the model of Ashcraft et al. (2007). Thus, both risk factors inadequate math skills and insufficient motivation might be strongly inter-related. Additionally, the less trained connections might also reflect the tendency to avoid working with numbers. Due to the low difficulty of the task the lower practice of working with numbers might solely be reflected in the more difficult trials (i.e., trials with small distance between the two numbers).

Moreover, we specifically investigated whether a deficient ANS (assessed using a non-symbolic dot comparison task) may be a risk factor according to the model of Ashcraft et al. (2007). However, we found that ANS acuity was not associated with math anxiety. Thus, our results did not provide evidence for the hypothesis that a deficient ANS might be a risk factor for the development of math anxiety. Similarly, our results do not support the hybrid model of Maloney et al. (2011) who postulate that a less precise ANS plays a role in the development of math anxiety, since we did not find a relationship between ANS acuity and math anxiety.

## Conclusion

Taken together, our findings question the previous conclusion that a less precise ANS is associated with higher math anxiety. Our results revealed that ANS acuity – when being measured by the standard ANS task (i.e., a non-symbolic dot comparison task) – was not associated with math anxiety at all. However, we replicated the association of the distance effect for symbolic number comparison and math anxiety. Thus, impaired processes in symbolic but not non-symbolic magnitude comparison seem to underlie math anxiety. Generally, this finding fits nicely in the model of Ashcraft et al. (2007), who proposed that inadequate basic numerical competencies constitute a risk factor for the development of math anxiety. According to our results this risk factor might also include impaired symbolic number comparison processes.

## Conflict of Interest Statement

The authors declare that the research was conducted in the absence of any commercial or financial relationships that could be construed as a potential conflict of interest.
