# Supplementary material for: The influence of math anxiety on symbolic and non-symbolic magnitude processing
Source: Front Psychol. 2015 Oct 27;6:1621. doi: 10.3389/fpsyg.2015.01621 (PMC4621307; doi:10.3389/fpsyg.2015.01621)
Supplement: Supplementary file 1 [file Data_Sheet_1.DOCX]

**Appendix**

We divided our sample in a low and a high math anxiety group by using a median split and repeated the analyses reported in the main article. The math anxiety groups were effect coded (high math anxiety +0.5, low math anxiety -0.5). The pattern of results was identical to the one described in the main article with math anxiety as a continuous predictor. We found significant effects of numerical distance and size for both response time (RT) and error rates (ER) in the symbolic and the non-symbolic comparison task. Importantly, we observed a reliable interaction between math anxiety and distance in the symbolic comparison task for RT. High math anxious individuals showed a larger distance effect than low math anxious individuals (high math anxiety: estimate = -17.80 ms vs. low math anxiety: estimate = -14.47 ms). However, we did not find a significant interaction between math anxiety and distance in the non-symbolic dot comparison task. Moreover, there was no significant interaction between size and math anxiety neither for the symbolic nor the non-symbolic comparison task. Additionally, there was also no main effect of math anxiety on RT or ER.

**Table A1.** Estimates of fixed effects (ms) for response times.

| **Task** | **Effect** | **Estimate (*SE*)** | ***df*** | ***t*** | ***p*** | **95% CI** |
| --- | --- | --- | --- | --- | --- | --- |
| Symbolic comparison |  |  |  |  |  |  |
|  | Intercept | 665.358 (9.99) | 61.00 | 66.61 | <.001 | [645.78, 684.94] |
|  | Distance | -16.139 (0.76) | 61.00 | -21.20 | <.001 | [-17.63, -14.65] |
|  | MA group | 36.055 (19.98) | 61.00 | 1.80 | .076 | [-3.10, 75.21] |
|  | Size | 4.160 (0.44) | 60.89 | 9.50 | <.001 | [3.30, 5.02] |
|  | Distance × MA group | -3.331 (1.52) | 61.00 | -2.19 | .033 | [-6.32, -0.35] |
|  | Size × MA group | 0.391 (0.88) | 60.89 | 0.45 | .657 | [-1.33, 2.11] |
| Non-symbolic comparison |  |  |  |  |  |  |
|  | Intercept | 681.825 (25.29) | 61.35 | 26.96 | <.001 | [632.26, 731.40] |
|  | Distance | -1.884 (0.57) | 93.30 | -3.29 | .001 | [-3.01, -0.76] |
|  | MA group | -27.307 (50.51) | 61.00 | -0.54 | .591 | [-126.31, 71.70] |
|  | Size | -1.119 (0.22) | 98.20 | -5.15 | <.001 | [-1.54, -0.69] |
|  | Distance × MA group | -0.978 (1.01) | 60.84 | -0.97 | .338 | [-2.96, 1.01] |
|  | Size × MA group | -0.220 (0.38) | 60.87 | -0.58 | .563 | [-0.96, 0.52] |

*Note*. 95% CI based on the estimated local curvature of the likelihood surface. Math anxiety groups were effect coded (high math anxiety +0.5, low math anxiety -0.5). MA = math anxiety.

**Table A2.** Estimates of fixed effects (log odds) for error rates.

| **Task** | **Effect** | **Estimate (*SE*)** | ***χ^2^*** | ***p*** | **95% CI** |
| --- | --- | --- | --- | --- | --- |
| Symbolic comparison |  |  |  |  |  |
|  | Intercept | -3.792 (0.107) | - | - | [-4.002, -3.581] |
|  | Distance | -0.350 (0.024) | 244.25 | <.001 | [-0.398, -0.303] |
|  | MA group | -0.011 (0.106) | 0.01 | .918 | [-0.219, 0.197] |
|  | Size | 0.148 (0.010) | 234.54 | <.001 | [0.128, 0.167] |
|  | Distance × MA group | 0.041 (0.024) | 2.81 | .093 | [-0.007, 0.089] |
|  | Size × MA group | -0.001 (0.010) | 0.01 | .903 | [-0.021, 0.018] |
| Non-symbolic comparison |  |  |  |  |  |
|  | Intercept | -0.839 (0.071) | - | - | [-0.979, -0.700] |
|  | Distance | -0.070 (0.008) | 70.17 | <.001 | [-0.085, -0.054] |
|  | MA group | 0.087 (0.121) | 0.52 | .472 | [-0.150, 0.325] |
|  | Size | 0.009 (0.003) | 7.25 | .007 | [0.002, 0.015] |
|  | Distance × MA group | 0.004 (0.006) | 0.52 | .471 | [-0.007, 0.016] |
|  | Size × MA group | 0.002 (0.002) | 1.05 | .305 | [-0.002, 0.007] |

*Note*. *p*-values were obtained via likelihood ratio tests (*df* =1). 95% CI are based on the estimated local curvature of the likelihood surface. Math anxiety groups were effect coded (high math anxiety +0.5, low math anxiety -0.5). MA = math anxiety.
